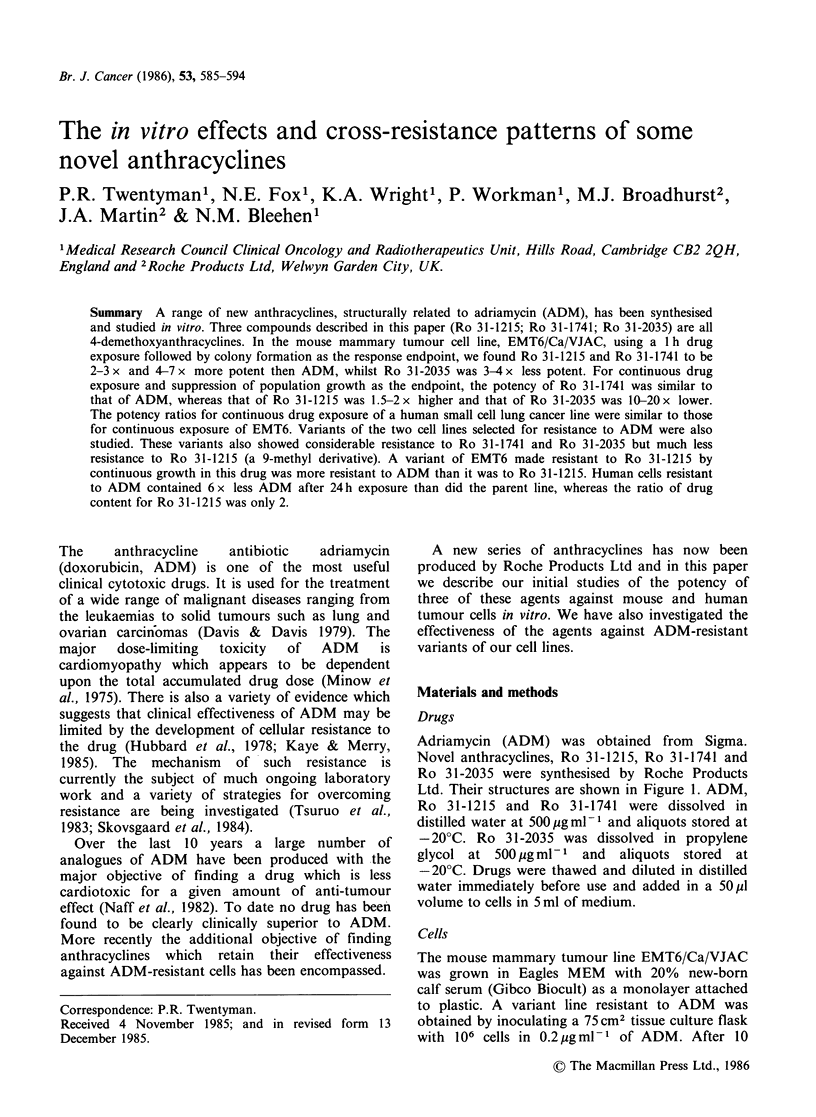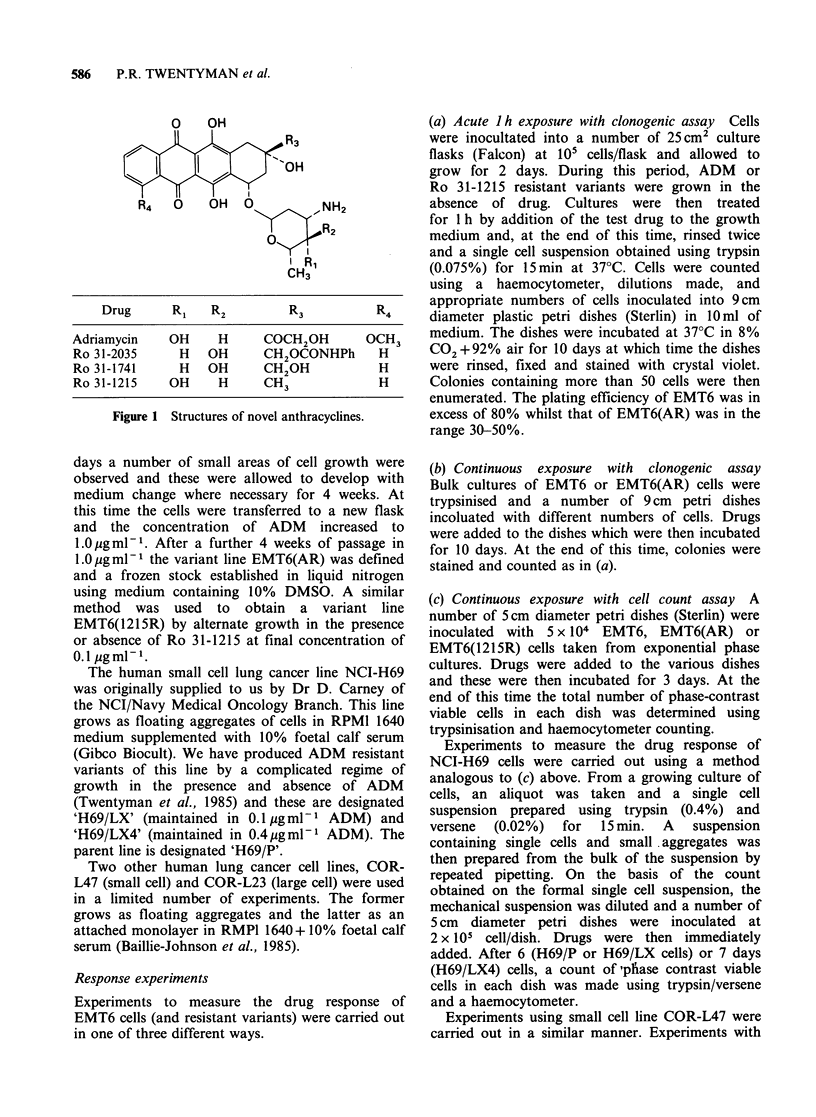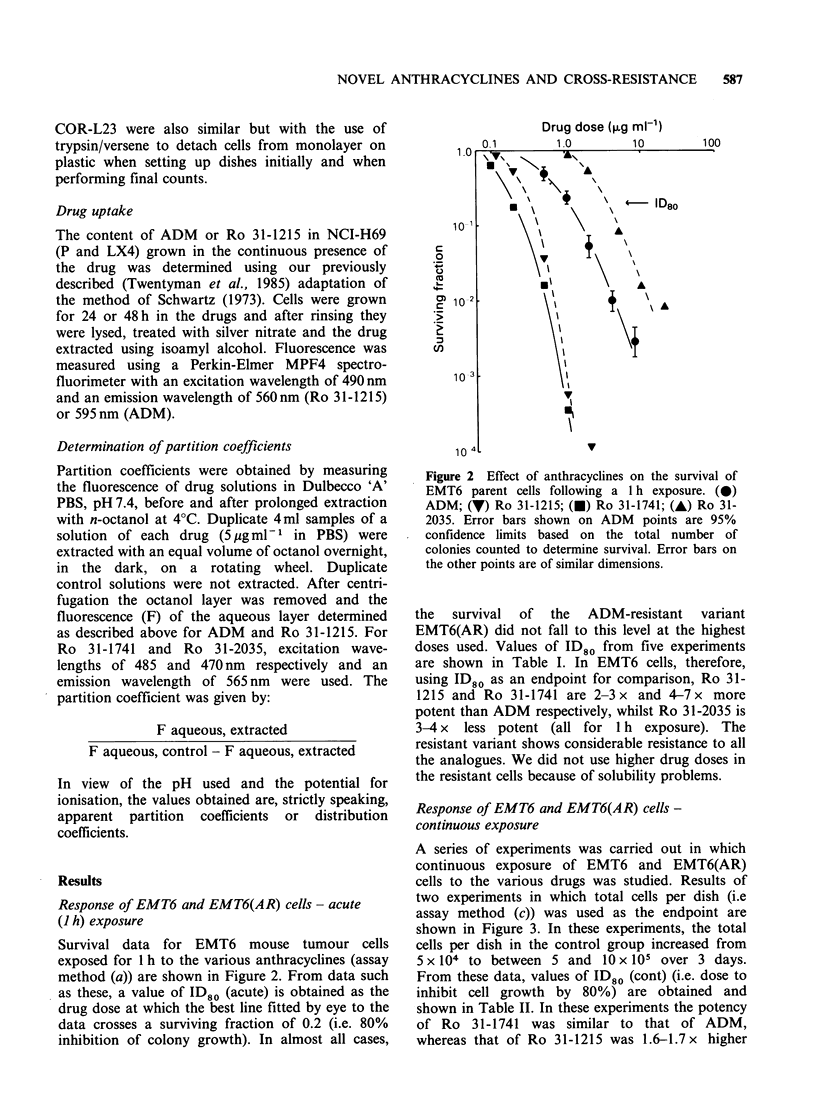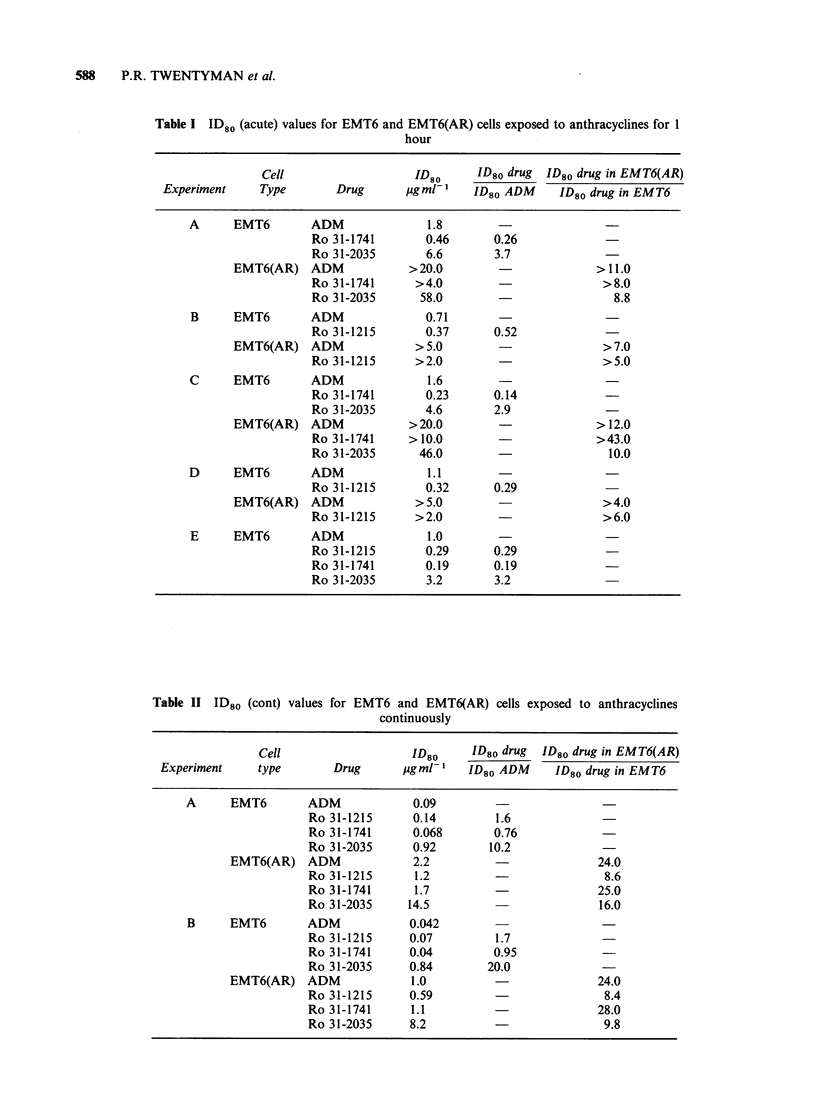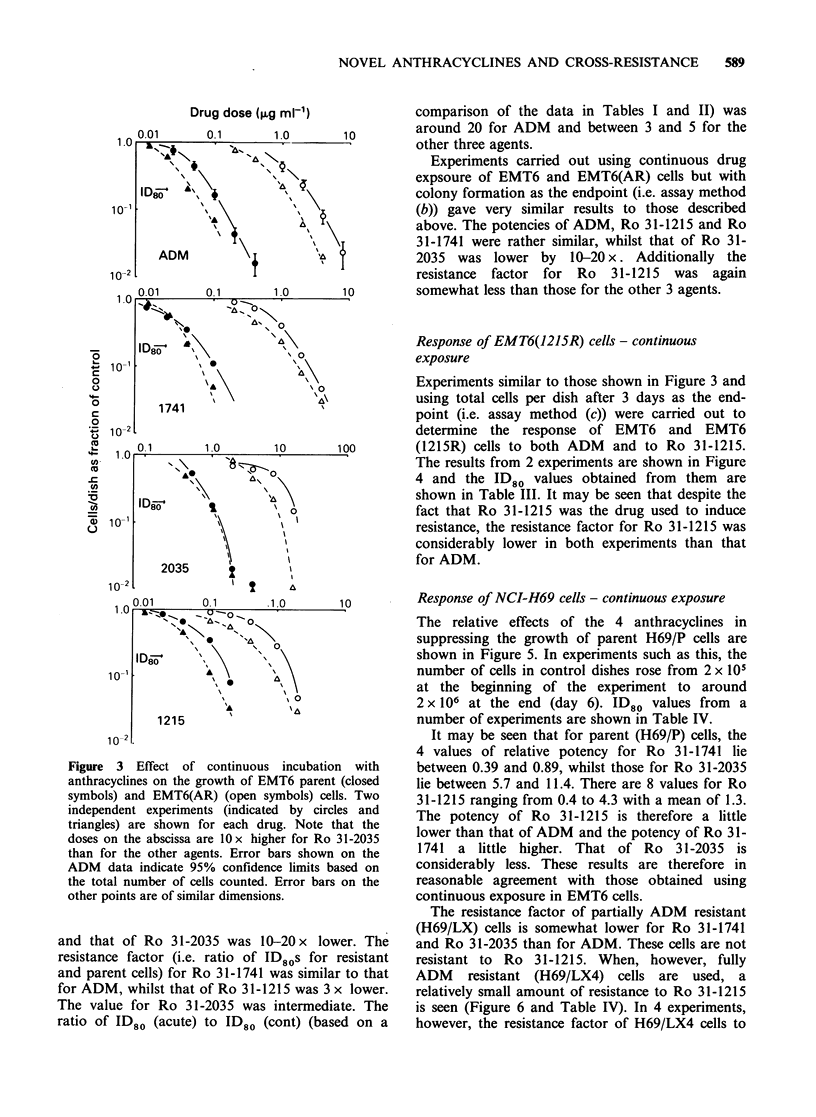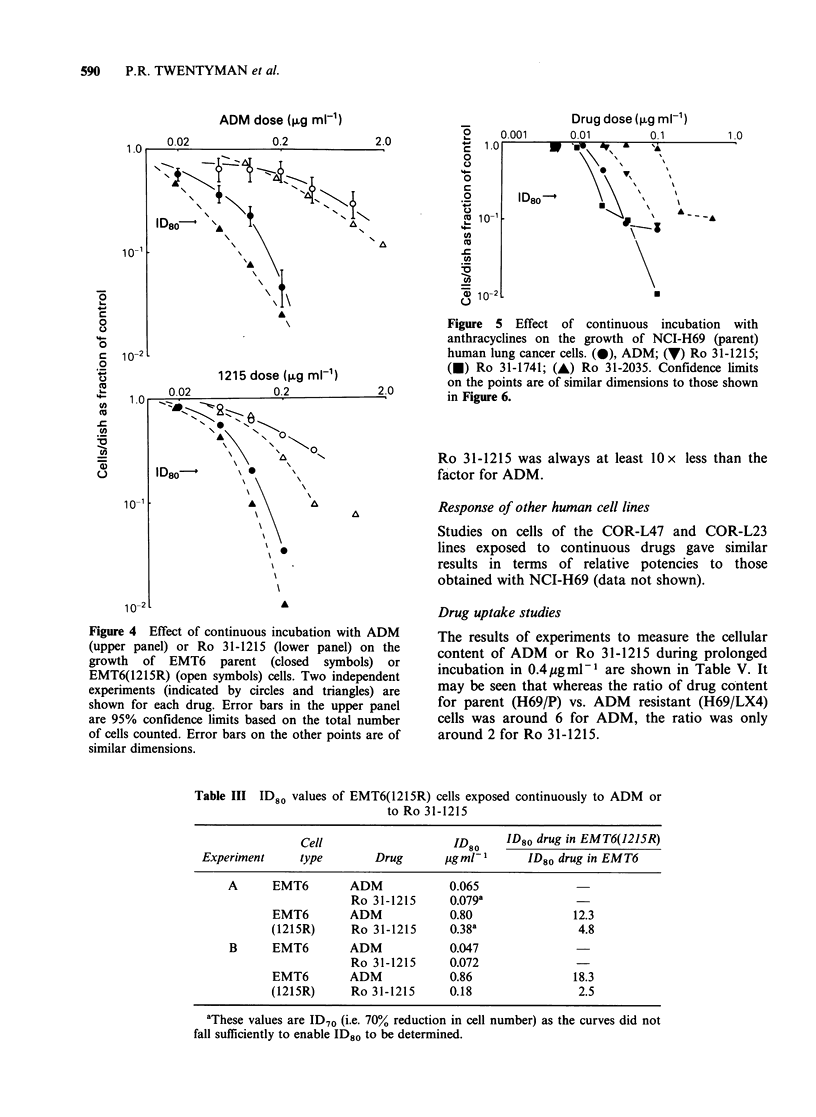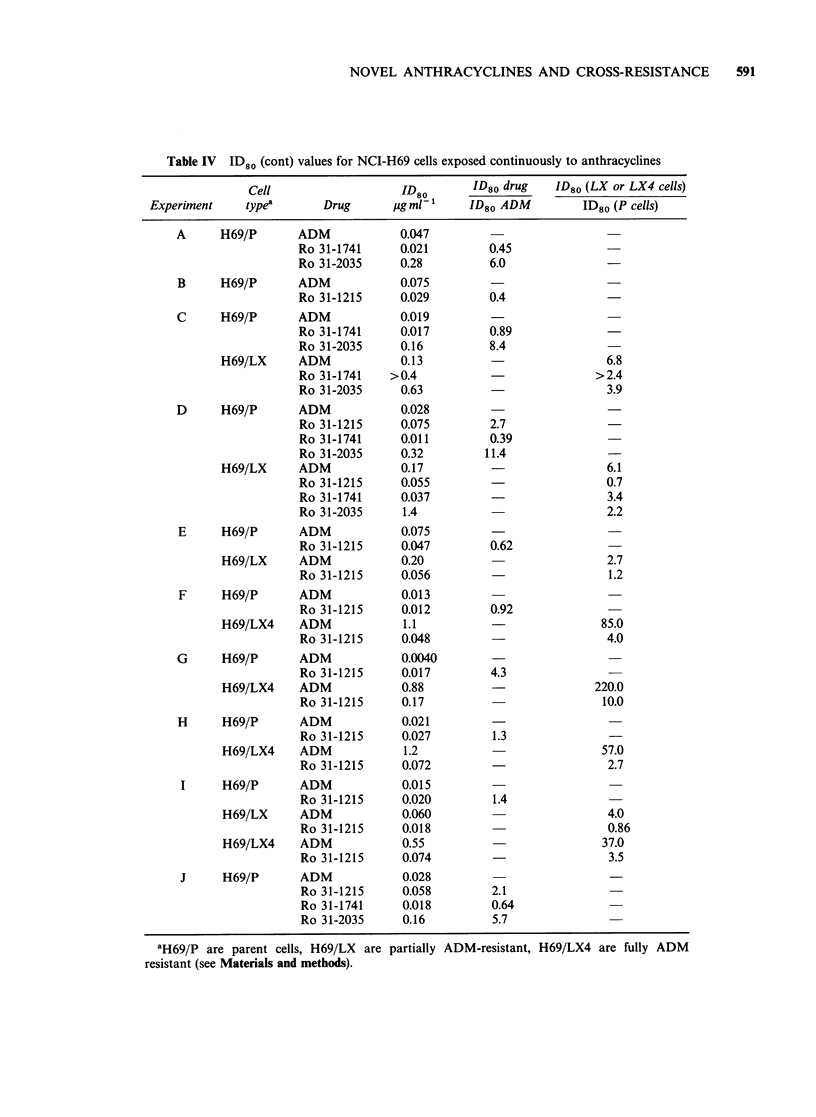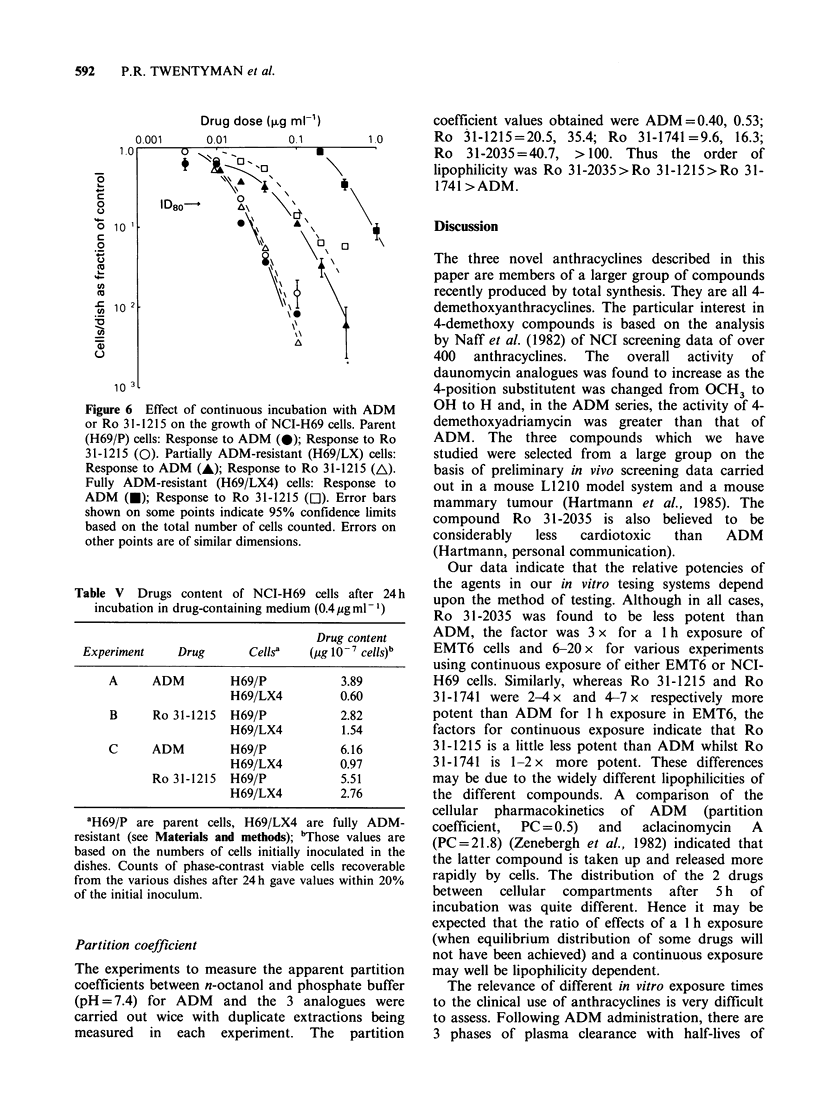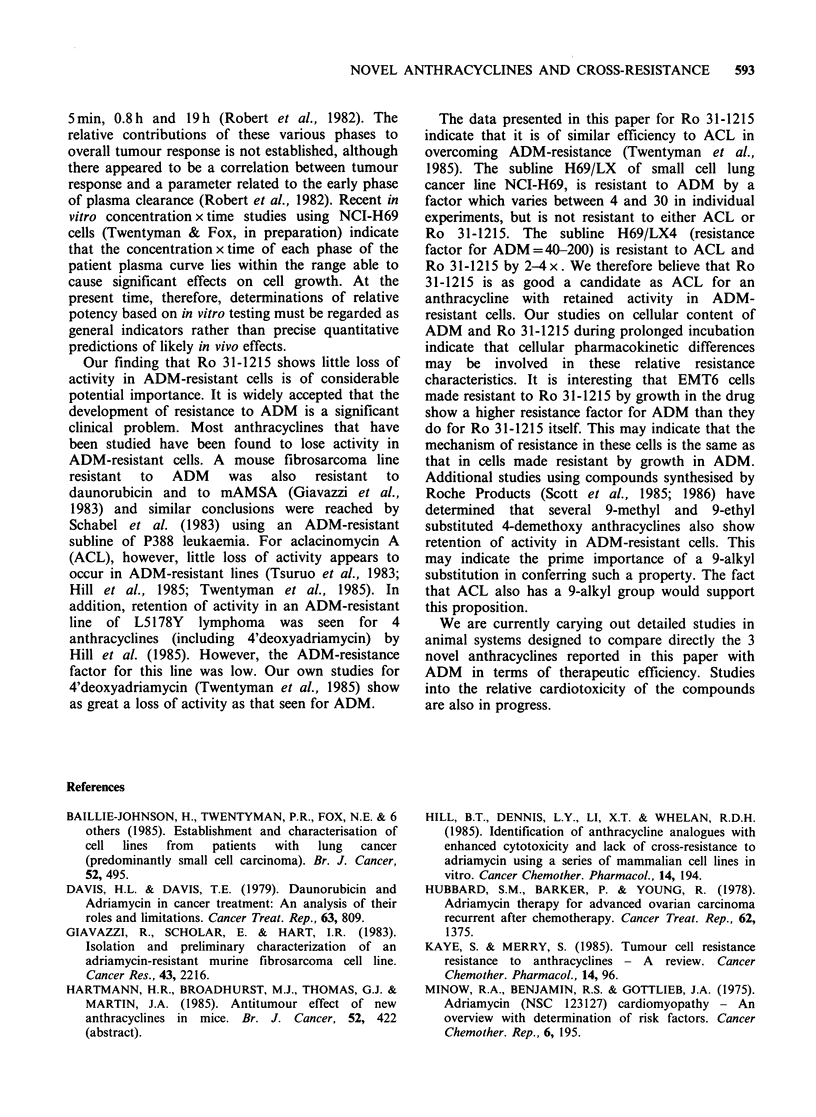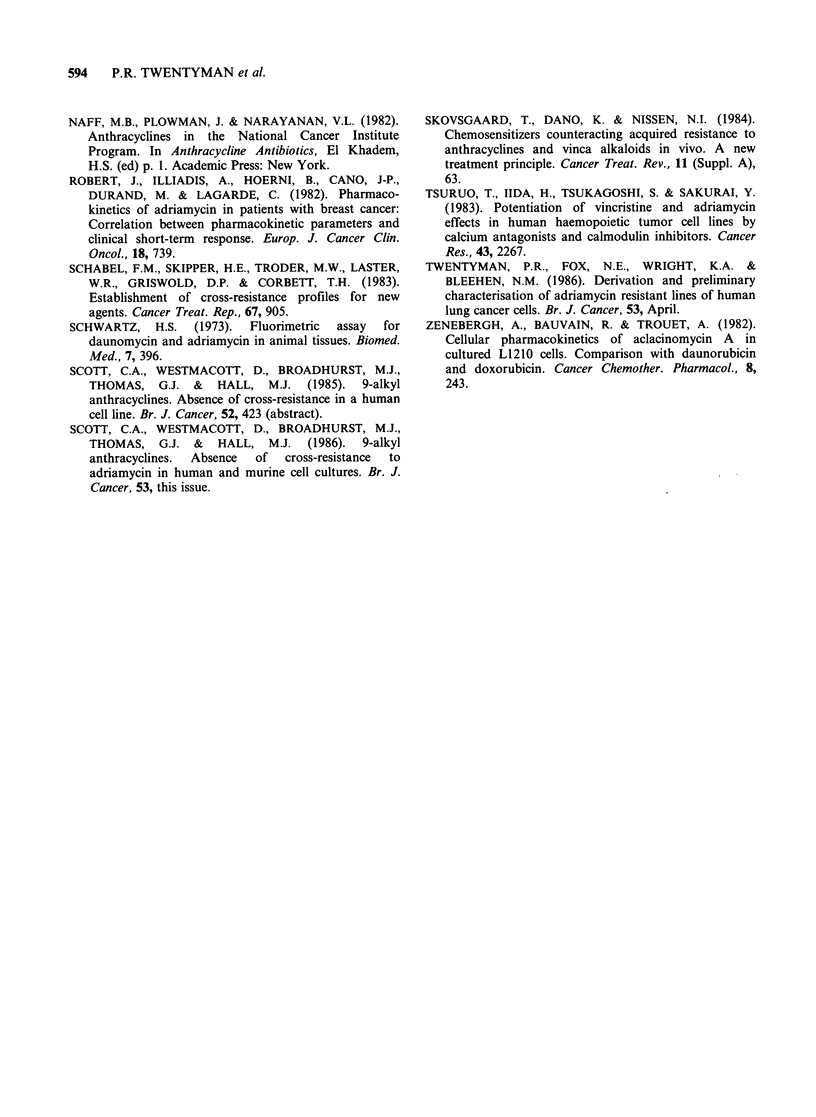# The in vitro effects and cross-resistance patterns of some novel anthracyclines.

**DOI:** 10.1038/bjc.1986.100

**Published:** 1986-05

**Authors:** P. R. Twentyman, N. E. Fox, K. A. Wright, P. Workman, M. J. Broadhurst, J. A. Martin, N. M. Bleehen

## Abstract

A range of new anthracyclines, structurally related to adriamycin (ADM), has been synthesised and studied in vitro. Three compounds described in this paper (Ro 31-1215; Ro 31-1741; Ro 31-2035) are all 4-demethoxyanthracyclines. In the mouse mammary tumour cell line, EMT6/Ca/VJAC, using a 1 h drug exposure followed by colony formation as the response endpoint, we found Ro 31-1215 and Ro 31-1741 to be 2-3 x and 4-7 x more potent then ADM, whilst Ro 31-2035 was 3-4 x less potent. For continuous drug exposure and suppression of population growth as the endpoint, the potency of Ro 31-1741 was similar to that of ADM, whereas that of Ro 31-1215 was 1.5-2 x higher and that of Ro 31-2035 was 10-20 x lower. The potency ratios for continuous drug exposure of a human small cell lung cancer line were similar to those for continuous exposure of EMT6. Variants of the two cell lines selected for resistance to ADM were also studied. These variants also showed considerable resistance to Ro 31-1741 and Ro 31-2035 but much less resistance to Ro 31-1215 (a 9-methyl derivative). A variant of EMT6 made resistant to Ro 31-1215 by continuous growth in this drug was more resistant to ADM than it was to Ro 31-1215. Human cells resistant to ADM contained 6 x less ADM after 24 h exposure than did the parent line, whereas the ratio of drug content for Ro 31-1215 was only 2.


Br. J. Cancer (1986), **53**, 585–594

### The *in vitro* effects and cross-resistance patterns of some novel anthracyclines

P.R. Twentyman[1], N.E. Fox[1], K.A. Wright[1], P. Workman[1], M.J. Broadhurst[2], J.A. Martin[2] & N.M. Bleehen[1]

[1]*Medical Research Council Clinical Oncology and Radiotherapeutics Unit, Hills Road, Cambridge CB2 2QH, England and* [2]*Roche Products Ltd, Welwyn Garden City, UK.*

**Summary** A range of new anthracyclines, structurally related to adriamycin (ADM), has been synthesised and studied *in vitro*. Three compounds described in this paper (Ro 31-1215; Ro 31-1741; Ro 31-2035) are all 4-demethoxyanthracyclines. In the mouse mammary tumour cell line, EMT6/Ca/VJAC, using a 1 h drug exposure followed by colony formation as the response endpoint, we found Ro 31-1215 and Ro 31-1741 to be 2–3× and 4–7× more potent then ADM, whilst Ro 31-2035 was 3–4× less potent. For continuous drug exposure and suppression of population growth as the endpoint, the potency of Ro 31-1741 was similar to that of ADM, whereas that of Ro 31-1215 was 1.5–2× higher and that of Ro 31-2035 was 10–20× lower. The potency ratios for continuous drug exposure of a human small cell lung cancer line were similar to those for continuous exposure of EMT6. Variants of the two cell lines selected for resistance to ADM were also studied. These variants also showed considerable resistance to Ro 31-1741 and Ro 31-2035 but much less resistance to Ro 31-1215 (a 9-methyl derivative). A variant of EMT6 made resistant to Ro 31-1215 by continuous growth in this drug was more resistant to ADM than it was to Ro 31-1215. Human cells resistant to ADM contained 6× less ADM after 24 h exposure than did the parent line, whereas the ratio of drug content for Ro 31-1215 was only 2.

The anthracycline antibiotic adriamycin (doxorubicin, ADM) is one of the most useful clinical cytotoxic drugs. It is used for the treatment of a wide range of malignant diseases ranging from the leukaemias to solid tumours such as lung and ovarian carcinomas (Davis & Davis 1979). The major dose-limiting toxicity of ADM is cardiomyopathy which appears to be dependent upon the total accumulated drug dose (Minow *et al.*, 1975). There is also a variety of evidence which suggests that clinical effectiveness of ADM may be limited by the development of cellular resistance to the drug (Hubbard *et al.*, 1978; Kaye & Merry, 1985). The mechanism of such resistance is currently the subject of much ongoing laboratory work and a variety of strategies for overcoming resistance are being investigated (Tsuruo *et al.*, 1983; Skovsgaard *et al.*, 1984).

Over the last 10 years a large number of analogues of ADM have been produced with the major objective of finding a drug which is less cardiotoxic for a given amount of anti-tumour effect (Naff *et al.*, 1982). To date no drug has been found to be clearly clinically superior to ADM. More recently the additional objective of finding anthracyclines which retain their effectiveness against ADM-resistant cells has been encompassed.

Correspondence: P.R. Twentyman.
Received 4 November 1985; and in revised form 13 December 1985.

A new series of anthracyclines has now been produced by Roche Products Ltd and in this paper we describe our initial studies of the potency of three of these agents against mouse and human tumour cells *in vitro*. We have also investigated the effectiveness of the agents against ADM-resistant variants of our cell lines.

### Materials and methods

### *Drugs*

Adriamycin (ADM) was obtained from Sigma. Novel anthracyclines, Ro 31-1215, Ro 31-1741 and Ro 31-2035 were synthesised by Roche Products Ltd. Their structures are shown in Figure 1. ADM, Ro 31-1215 and Ro 31-1741 were dissolved in distilled water at $500\,\mu g\,ml^{-1}$ and aliquots stored at $-20°C$. Ro 31-2035 was dissolved in propylene glycol at $500\,\mu g\,ml^{-1}$ and aliquots stored at $-20°C$. Drugs were thawed and diluted in distilled water immediately before use and added in a $50\,\mu l$ volume to cells in 5 ml of medium.

### *Cells*

The mouse mammary tumour line EMT6/Ca/VJAC was grown in Eagles MEM with 20% new-born calf serum (Gibco Biocult) as a monolayer attached to plastic. A variant line resistant to ADM was obtained by inoculating a $75\,cm^2$ tissue culture flask with $10^6$ cells in $0.2\,\mu g\,ml^{-1}$ of ADM. After 10

© The Macmillan Press Ltd., 1986

| Drug | $R_1$ | $R_2$ | $R_3$ | $R_4$ |
|---|---|---|---|---|
| Adriamycin | OH | H | $COCH_2OH$ | $OCH_3$ |
| Ro 31-2035 | H | OH | $CH_2OCONHPh$ | H |
| Ro 31-1741 | H | OH | $CH_2OH$ | H |
| Ro 31-1215 | OH | H | $CH_3$ | H |

**Figure 1** Structures of novel anthracyclines.

days a number of small areas of cell growth were observed and these were allowed to develop with medium change where necessary for 4 weeks. At this time the cells were transferred to a new flask and the concentration of ADM increased to $1.0\,\mu g\,ml^{-1}$. After a further 4 weeks of passage in $1.0\,\mu g\,ml^{-1}$ the variant line EMT6(AR) was defined and a frozen stock established in liquid nitrogen using medium containing 10% DMSO. A similar method was used to obtain a variant line EMT6(1215R) by alternate growth in the presence or absence of Ro 31-1215 at final concentration of $0.1\,\mu g\,ml^{-1}$.

The human small cell lung cancer line NCI-H69 was originally supplied to us by Dr D. Carney of the NCI/Navy Medical Oncology Branch. This line grows as floating aggregates of cells in RPMI 1640 medium supplemented with 10% foetal calf serum (Gibco Biocult). We have produced ADM resistant variants of this line by a complicated regime of growth in the presence and absence of ADM (Twentyman *et al.*, 1985) and these are designated 'H69/LX' (maintained in $0.1\,\mu g\,ml^{-1}$ ADM) and 'H69/LX4' (maintained in $0.4\,\mu g\,ml^{-1}$ ADM). The parent line is designated 'H69/P'.

Two other human lung cancer cell lines, COR-L47 (small cell) and COR-L23 (large cell) were used in a limited number of experiments. The former grows as floating aggregates and the latter as an attached monolayer in RMPI 1640+10% foetal calf serum (Baillie-Johnson *et al.*, 1985).

*Response experiments*

Experiments to measure the drug response of EMT6 cells (and resistant variants) were carried out in one of three different ways.

(*a*) *Acute 1 h exposure with clonogenic assay* Cells were inocultated into a number of $25\,cm^2$ culture flasks (Falcon) at $10^5$ cells/flask and allowed to grow for 2 days. During this period, ADM or Ro 31-1215 resistant variants were grown in the absence of drug. Cultures were then treated for 1 h by addition of the test drug to the growth medium and, at the end of this time, rinsed twice and a single cell suspension obtained using trypsin (0.075%) for 15 min at 37°C. Cells were counted using a haemocytometer, dilutions made, and appropriate numbers of cells inoculated into 9 cm diameter plastic petri dishes (Sterlin) in 10 ml of medium. The dishes were incubated at 37°C in 8% $CO_2$ +92% air for 10 days at which time the dishes were rinsed, fixed and stained with crystal violet. Colonies containing more than 50 cells were then enumerated. The plating efficiency of EMT6 was in excess of 80% whilst that of EMT6(AR) was in the range 30–50%.

(*b*) *Continuous exposure with clonogenic assay* Bulk cultures of EMT6 or EMT6(AR) cells were trypsinised and a number of 9 cm petri dishes incoluated with different numbers of cells. Drugs were added to the dishes which were then incubated for 10 days. At the end of this time, colonies were stained and counted as in (*a*).

(*c*) *Continuous exposure with cell count assay* A number of 5 cm diameter petri dishes (Sterlin) were inoculated with $5\times10^4$ EMT6, EMT6(AR) or EMT6(1215R) cells taken from exponential phase cultures. Drugs were added to the various dishes and these were then incubated for 3 days. At the end of this time the total number of phase-contrast viable cells in each dish was determined using trypsinisation and haemocytometer counting.

Experiments to measure the drug response of NCI-H69 cells were carried out using a method analogous to (*c*) above. From a growing culture of cells, an aliquot was taken and a single cell suspension prepared using trypsin (0.4%) and versene (0.02%) for 15 min. A suspension containing single cells and small aggregates was then prepared from the bulk of the suspension by repeated pipetting. On the basis of the count obtained on the formal single cell suspension, the mechanical suspension was diluted and a number of 5 cm diameter petri dishes were inoculated at $2\times10^5$ cell/dish. Drugs were then immediately added. After 6 (H69/P or H69/LX cells) or 7 days (H69/LX4) cells, a count of phase contrast viable cells in each dish was made using trypsin/versene and a haemocytometer.

Experiments using small cell line COR-L47 were carried out in a similar manner. Experiments with

COR-L23 were also similar but with the use of trypsin/versene to detach cells from monolayer on plastic when setting up dishes initially and when performing final counts.

*Drug uptake*

The content of ADM or Ro 31-1215 in NCI-H69 (P and LX4) grown in the continuous presence of the drug was determined using our previously described (Twentyman *et al.*, 1985) adaptation of the method of Schwartz (1973). Cells were grown for 24 or 48 h in the drugs and after rinsing they were lysed, treated with silver nitrate and the drug extracted using isoamyl alcohol. Fluorescence was measured using a Perkin-Elmer MPF4 spectrofluorimeter with an excitation wavelength of 490 nm and an emission wavelength of 560 nm (Ro 31-1215) or 595 nm (ADM).

*Determination of partition coefficients*

Partition coefficients were obtained by measuring the fluorescence of drug solutions in Dulbecco 'A' PBS, pH 7.4, before and after prolonged extraction with *n*-octanol at 4°C. Duplicate 4 ml samples of a solution of each drug ($5\,\mu g\,ml^{-1}$ in PBS) were extracted with an equal volume of octanol overnight, in the dark, on a rotating wheel. Duplicate control solutions were not extracted. After centrifugation the octanol layer was removed and the fluorescence (F) of the aqueous layer determined as described above for ADM and Ro 31-1215. For Ro 31-1741 and Ro 31-2035, excitation wavelengths of 485 and 470 nm respectively and an emission wavelength of 565 nm were used. The partition coefficient was given by:

$$\frac{\text{F aqueous, extracted}}{\text{F aqueous, control} - \text{F aqueous, extracted}}$$

In view of the pH used and the potential for ionisation, the values obtained are, strictly speaking, apparent partition coefficients or distribution coefficients.

### Results

*Response of EMT6 and EMT6(AR) cells – acute (1 h) exposure*

Survival data for EMT6 mouse tumour cells exposed for 1 h to the various anthracyclines (assay method (*a*)) are shown in Figure 2. From data such as these, a value of $ID_{80}$ (acute) is obtained as the drug dose at which the best line fitted by eye to the data crosses a surviving fraction of 0.2 (i.e. 80% inhibition of colony growth). In almost all cases,

**Figure 2** Effect of anthracyclines on the survival of EMT6 parent cells following a 1 h exposure. (●) ADM; (▼) Ro 31-1215; (■) Ro 31-1741; (▲) Ro 31-2035. Error bars shown on ADM points are 95% confidence limits based on the total number of colonies counted to determine survival. Error bars on the other points are of similar dimensions.

the survival of the ADM-resistant variant EMT6(AR) did not fall to this level at the highest doses used. Values of $ID_{80}$ from five experiments are shown in Table I. In EMT6 cells, therefore, using $ID_{80}$ as an endpoint for comparison, Ro 31-1215 and Ro 31-1741 are 2–3 × and 4–7 × more potent than ADM respectively, whilst Ro 31-2035 is 3–4 × less potent (all for 1 h exposure). The resistant variant shows considerable resistance to all the analogues. We did not use higher drug doses in the resistant cells because of solubility problems.

*Response of EMT6 and EMT6(AR) cells – continuous exposure*

A series of experiments was carried out in which continuous exposure of EMT6 and EMT6(AR) cells to the various drugs was studied. Results of two experiments in which total cells per dish (i.e assay method (*c*)) was used as the endpoint are shown in Figure 3. In these experiments, the total cells per dish in the control group increased from $5 \times 10^4$ to between 5 and $10 \times 10^5$ over 3 days. From these data, values of $ID_{80}$ (cont) (i.e. dose to inhibit cell growth by 80%) are obtained and shown in Table II. In these experiments the potency of Ro 31-1741 was similar to that of ADM, whereas that of Ro 31-1215 was 1.6–1.7 × higher

**Table I** $ID_{80}$ (acute) values for EMT6 and EMT6(AR) cells exposed to anthracyclines for 1 hour

| Experiment | Cell Type | Drug | $ID_{80}$ $\mu g\,ml^{-1}$ | $\frac{ID_{80}\ drug}{ID_{80}\ ADM}$ | $\frac{ID_{80}\ drug\ in\ EMT6(AR)}{ID_{80}\ drug\ in\ EMT6}$ |
|---|---|---|---|---|---|
| A | EMT6 | ADM | 1.8 | — | — |
| | | Ro 31-1741 | 0.46 | 0.26 | — |
| | | Ro 31-2035 | 6.6 | 3.7 | — |
| | EMT6(AR) | ADM | >20.0 | — | >11.0 |
| | | Ro 31-1741 | >4.0 | — | >8.0 |
| | | Ro 31-2035 | 58.0 | — | 8.8 |
| B | EMT6 | ADM | 0.71 | — | — |
| | | Ro 31-1215 | 0.37 | 0.52 | — |
| | EMT6(AR) | ADM | >5.0 | — | >7.0 |
| | | Ro 31-1215 | >2.0 | — | >5.0 |
| C | EMT6 | ADM | 1.6 | — | — |
| | | Ro 31-1741 | 0.23 | 0.14 | — |
| | | Ro 31-2035 | 4.6 | 2.9 | — |
| | EMT6(AR) | ADM | >20.0 | — | >12.0 |
| | | Ro 31-1741 | >10.0 | — | >43.0 |
| | | Ro 31-2035 | 46.0 | — | 10.0 |
| D | EMT6 | ADM | 1.1 | — | — |
| | | Ro 31-1215 | 0.32 | 0.29 | — |
| | EMT6(AR) | ADM | >5.0 | — | >4.0 |
| | | Ro 31-1215 | >2.0 | — | >6.0 |
| E | EMT6 | ADM | 1.0 | — | — |
| | | Ro 31-1215 | 0.29 | 0.29 | — |
| | | Ro 31-1741 | 0.19 | 0.19 | — |
| | | Ro 31-2035 | 3.2 | 3.2 | — |

**Table II** $ID_{80}$ (cont) values for EMT6 and EMT6(AR) cells exposed to anthracyclines continuously

| Experiment | Cell type | Drug | $ID_{80}$ $\mu g\,ml^{-1}$ | $\frac{ID_{80}\ drug}{ID_{80}\ ADM}$ | $\frac{ID_{80}\ drug\ in\ EMT6(AR)}{ID_{80}\ drug\ in\ EMT6}$ |
|---|---|---|---|---|---|
| A | EMT6 | ADM | 0.09 | — | — |
| | | Ro 31-1215 | 0.14 | 1.6 | — |
| | | Ro 31-1741 | 0.068 | 0.76 | — |
| | | Ro 31-2035 | 0.92 | 10.2 | — |
| | EMT6(AR) | ADM | 2.2 | — | 24.0 |
| | | Ro 31-1215 | 1.2 | — | 8.6 |
| | | Ro 31-1741 | 1.7 | — | 25.0 |
| | | Ro 31-2035 | 14.5 | — | 16.0 |
| B | EMT6 | ADM | 0.042 | — | — |
| | | Ro 31-1215 | 0.07 | 1.7 | — |
| | | Ro 31-1741 | 0.04 | 0.95 | — |
| | | Ro 31-2035 | 0.84 | 20.0 | — |
| | EMT6(AR) | ADM | 1.0 | — | 24.0 |
| | | Ro 31-1215 | 0.59 | — | 8.4 |
| | | Ro 31-1741 | 1.1 | — | 28.0 |
| | | Ro 31-2035 | 8.2 | — | 9.8 |

**Figure 3** Effect of continuous incubation with anthracyclines on the growth of EMT6 parent (closed symbols) and EMT6(AR) (open symbols) cells. Two independent experiments (indicated by circles and triangles) are shown for each drug. Note that the doses on the abscissa are 10 x higher for Ro 31-2035 than for the other agents. Error bars shown on the ADM data indicate 95% confidence limits based on the total number of cells counted. Error bars on the other points are of similar dimensions.

and that of Ro 31-2035 was 10–20 x lower. The resistance factor (i.e. ratio of $ID_{80}$s for resistant and parent cells) for Ro 31-1741 was similar to that for ADM, whilst that of Ro 31-1215 was 3 x lower. The value for Ro 31-2035 was intermediate. The ratio of $ID_{80}$ (acute) to $ID_{80}$ (cont) (based on a comparison of the data in Tables I and II) was around 20 for ADM and between 3 and 5 for the other three agents.

Experiments carried out using continuous drug expsoure of EMT6 and EMT6(AR) cells but with colony formation as the endpoint (i.e. assay method (*b*)) gave very similar results to those described above. The potencies of ADM, Ro 31-1215 and Ro 31-1741 were rather similar, whilst that of Ro 31-2035 was lower by 10–20 x. Additionally the resistance factor for Ro 31-1215 was again somewhat less than those for the other 3 agents.

*Response of EMT6(1215R) cells – continuous exposure*

Experiments similar to those shown in Figure 3 and using total cells per dish after 3 days as the end-point (i.e. assay method (*c*)) were carried out to determine the response of EMT6 and EMT6 (1215R) cells to both ADM and to Ro 31-1215. The results from 2 experiments are shown in Figure 4 and the $ID_{80}$ values obtained from them are shown in Table III. It may be seen that despite the fact that Ro 31-1215 was the drug used to induce resistance, the resistance factor for Ro 31-1215 was considerably lower in both experiments than that for ADM.

*Response of NCI-H69 cells – continuous exposure*

The relative effects of the 4 anthracyclines in suppressing the growth of parent H69/P cells are shown in Figure 5. In experiments such as this, the number of cells in control dishes rose from $2 \times 10^5$ at the beginning of the experiment to around $2 \times 10^6$ at the end (day 6). $ID_{80}$ values from a number of experiments are shown in Table IV.

It may be seen that for parent (H69/P) cells, the 4 values of relative potency for Ro 31-1741 lie between 0.39 and 0.89, whilst those for Ro 31-2035 lie between 5.7 and 11.4. There are 8 values for Ro 31-1215 ranging from 0.4 to 4.3 with a mean of 1.3. The potency of Ro 31-1215 is therefore a little lower than that of ADM and the potency of Ro 31-1741 a little higher. That of Ro 31-2035 is considerably less. These results are therefore in reasonable agreement with those obtained using continuous exposure in EMT6 cells.

The resistance factor of partially ADM resistant (H69/LX) cells is somewhat lower for Ro 31-1741 and Ro 31-2035 than for ADM. These cells are not resistant to Ro 31-1215. When, however, fully ADM resistant (H69/LX4) cells are used, a relatively small amount of resistance to Ro 31-1215 is seen (Figure 6 and Table IV). In 4 experiments, however, the resistance factor of H69/LX4 cells to

Figure 4 Effect of continuous incubation with ADM (upper panel) or Ro 31-1215 (lower panel) on the growth of EMT6 parent (closed symbols) or EMT6(1215R) (open symbols) cells. Two independent experiments (indicated by circles and triangles) are shown for each drug. Error bars in the upper panel are 95% confidence limits based on the total number of cells counted. Error bars on the other points are of similar dimensions.

**Figure 5** Effect of continuous incubation with anthracyclines on the growth of NCI-H69 (parent) human lung cancer cells. (●), ADM; (▼) Ro 31-1215; (■) Ro 31-1741; (▲) Ro 31-2035. Confidence limits on the points are of similar dimensions to those shown in **Figure 6.**

Ro 31-1215 was always at least 10× less than the factor for ADM.

*Response of other human cell lines*

Studies on cells of the COR-L47 and COR-L23 lines exposed to continuous drugs gave similar results in terms of relative potencies to those obtained with NCI-H69 (data not shown).

*Drug uptake studies*

The results of experiments to measure the cellular content of ADM or Ro 31-1215 during prolonged incubation in $0.4 \mu g ml^{-1}$ are shown in Table V. It may be seen that whereas the ratio of drug content for parent (H69/P) vs. ADM resistant (H69/LX4) cells was around 6 for ADM, the ratio was only around 2 for Ro 31-1215.

**Table III** $ID_{80}$ values of EMT6(1215R) cells exposed continuously to ADM or to Ro 31-1215

| *Experiment* | *Cell type* | *Drug* | $ID_{80}$ $\mu g ml^{-1}$ | $ID_{80}$ drug in EMT6(1215R) / $ID_{80}$ drug in EMT6 |
|---|---|---|---|---|
| A | EMT6 | ADM | 0.065 | — |
| | | Ro 31-1215 | 0.079[a] | — |
| | EMT6 | ADM | 0.80 | 12.3 |
| | (1215R) | Ro 31-1215 | 0.38[a] | 4.8 |
| B | EMT6 | ADM | 0.047 | — |
| | | Ro 31-1215 | 0.072 | — |
| | EMT6 | ADM | 0.86 | 18.3 |
| | (1215R) | Ro 31-1215 | 0.18 | 2.5 |

[a]These values are $ID_{70}$ (i.e. 70% reduction in cell number) as the curves did not fall sufficiently to enable $ID_{80}$ to be determined.

**Table IV** $ID_{80}$ (cont) values for NCI-H69 cells exposed continuously to anthracyclines

| *Experiment* | *Cell type*[a] | *Drug* | $ID_{80}$ $\mu g\,ml^{-1}$ | $ID_{80}$ drug / $ID_{80}$ ADM | $ID_{80}$ (LX or LX4 cells) / $ID_{80}$ (P cells) |
|---|---|---|---|---|---|
| A | H69/P | ADM | 0.047 | — | — |
| | | Ro 31-1741 | 0.021 | 0.45 | — |
| | | Ro 31-2035 | 0.28 | 6.0 | — |
| B | H69/P | ADM | 0.075 | — | — |
| | | Ro 31-1215 | 0.029 | 0.4 | — |
| C | H69/P | ADM | 0.019 | — | — |
| | | Ro 31-1741 | 0.017 | 0.89 | — |
| | | Ro 31-2035 | 0.16 | 8.4 | — |
| | H69/LX | ADM | 0.13 | — | 6.8 |
| | | Ro 31-1741 | >0.4 | — | >2.4 |
| | | Ro 31-2035 | 0.63 | — | 3.9 |
| D | H69/P | ADM | 0.028 | — | — |
| | | Ro 31-1215 | 0.075 | 2.7 | — |
| | | Ro 31-1741 | 0.011 | 0.39 | — |
| | | Ro 31-2035 | 0.32 | 11.4 | — |
| | H69/LX | ADM | 0.17 | — | 6.1 |
| | | Ro 31-1215 | 0.055 | — | 0.7 |
| | | Ro 31-1741 | 0.037 | — | 3.4 |
| | | Ro 31-2035 | 1.4 | — | 2.2 |
| E | H69/P | ADM | 0.075 | — | — |
| | | Ro 31-1215 | 0.047 | 0.62 | — |
| | H69/LX | ADM | 0.20 | — | 2.7 |
| | | Ro 31-1215 | 0.056 | — | 1.2 |
| F | H69/P | ADM | 0.013 | — | — |
| | | Ro 31-1215 | 0.012 | 0.92 | — |
| | H69/LX4 | ADM | 1.1 | — | 85.0 |
| | | Ro 31-1215 | 0.048 | — | 4.0 |
| G | H69/P | ADM | 0.0040 | — | — |
| | | Ro 31-1215 | 0.017 | 4.3 | — |
| | H69/LX4 | ADM | 0.88 | — | 220.0 |
| | | Ro 31-1215 | 0.17 | — | 10.0 |
| H | H69/P | ADM | 0.021 | — | — |
| | | Ro 31-1215 | 0.027 | 1.3 | — |
| | H69/LX4 | ADM | 1.2 | — | 57.0 |
| | | Ro 31-1215 | 0.072 | — | 2.7 |
| I | H69/P | ADM | 0.015 | — | — |
| | | Ro 31-1215 | 0.020 | 1.4 | — |
| | H69/LX | ADM | 0.060 | — | 4.0 |
| | | Ro 31-1215 | 0.018 | — | 0.86 |
| | H69/LX4 | ADM | 0.55 | — | 37.0 |
| | | Ro 31-1215 | 0.074 | — | 3.5 |
| J | H69/P | ADM | 0.028 | — | — |
| | | Ro 31-1215 | 0.058 | 2.1 | — |
| | | Ro 31-1741 | 0.018 | 0.64 | — |
| | | Ro 31-2035 | 0.16 | 5.7 | — |

[a]H69/P are parent cells, H69/LX are partially ADM-resistant, H69/LX4 are fully ADM resistant (see **Materials and methods**).

**Figure 6** Effect of continuous incubation with ADM or Ro 31-1215 on the growth of NCI-H69 cells. Parent (H69/P) cells: Response to ADM (●); Response to Ro 31-1215 (○). Partially ADM-resistant (H69/LX) cells: Response to ADM (▲); Response to Ro 31-1215 (△). Fully ADM-resistant (H69/LX4) cells: Response to ADM (■); Response to Ro 31-1215 (□). Error bars shown on some points indicate 95% confidence limits based on the total number of cells counted. Errors on other points are of similar dimensions.

**Table V** Drugs content of NCI-H69 cells after 24h incubation in drug-containing medium (0.4 $\mu$g ml$^{-1}$)

| *Experiment* | *Drug* | *Cells*[a] | *Drug content* ($\mu$g $10^{-7}$ *cells*)[b] |
|---|---|---|---|
| A | ADM | H69/P | 3.89 |
| | | H69/LX4 | 0.60 |
| B | Ro 31-1215 | H69/P | 2.82 |
| | | H69/LX4 | 1.54 |
| C | ADM | H69/P | 6.16 |
| | | H69/LX4 | 0.97 |
| | Ro 31-1215 | H69/P | 5.51 |
| | | H69/LX4 | 2.76 |

[a]H69/P are parent cells, H69/LX4 are fully ADM-resistant (see **Materials and methods**); [b]Those values are based on the numbers of cells initially inoculated in the dishes. Counts of phase-contrast viable cells recoverable from the various dishes after 24 h gave values within 20% of the initial inoculum.

*Partition coefficient*

The experiments to measure the apparent partition coefficients between *n*-octanol and phosphate buffer (pH = 7.4) for ADM and the 3 analogues were carried out wice with duplicate extractions being measured in each experiment. The partition coefficient values obtained were ADM = 0.40, 0.53; Ro 31-1215 = 20.5, 35.4; Ro 31-1741 = 9.6, 16.3; Ro 31-2035 = 40.7, > 100. Thus the order of lipophilicity was Ro 31-2035 > Ro 31-1215 > Ro 31-1741 > ADM.

### Discussion

The three novel anthracyclines described in this paper are members of a larger group of compounds recently produced by total synthesis. They are all 4-demethoxyanthracyclines. The particular interest in 4-demethoxy compounds is based on the analysis by Naff *et al.* (1982) of NCI screening data of over 400 anthracyclines. The overall activity of daunomycin analogues was found to increase as the 4-position substitutent was changed from $OCH_3$ to OH to H and, in the ADM series, the activity of 4-demethoxyadriamycin was greater than that of ADM. The three compounds which we have studied were selected from a large group on the basis of preliminary *in vivo* screening data carried out in a mouse L1210 model system and a mouse mammary tumour (Hartmann *et al.*, 1985). The compound Ro 31-2035 is also believed to be considerably less cardiotoxic than ADM (Hartmann, personal communication).

Our data indicate that the relative potencies of the agents in our *in vitro* tesing systems depend upon the method of testing. Although in all cases, Ro 31-2035 was found to be less potent than ADM, the factor was 3× for a 1 h exposure of EMT6 cells and 6–20× for various experiments using continuous exposure of either EMT6 or NCI-H69 cells. Similarly, whereas Ro 31-1215 and Ro 31-1741 were 2–4× and 4–7× respectively more potent than ADM for 1 h exposure in EMT6, the factors for continuous exposure indicate that Ro 31-1215 is a little less potent than ADM whilst Ro 31-1741 is 1–2× more potent. These differences may be due to the widely different lipophilicities of the different compounds. A comparison of the cellular pharmacokinetics of ADM (partition coefficient, PC = 0.5) and aclacinomycin A (PC = 21.8) (Zenebergh *et al.*, 1982) indicated that the latter compound is taken up and released more rapidly by cells. The distribution of the 2 drugs between cellular compartments after 5 h of incubation was quite different. Hence it may be expected that the ratio of effects of a 1 h exposure (when equilibrium distribution of some drugs will not have been achieved) and a continuous exposure may well be lipophilicity dependent.

The relevance of different *in vitro* exposure times to the clinical use of anthracyclines is very difficult to assess. Following ADM administration, there are 3 phases of plasma clearance with half-lives of

5 min, 0.8 h and 19 h (Robert *et al.*, 1982). The relative contributions of these various phases to overall tumour response is not established, although there appeared to be a correlation between tumour response and a parameter related to the early phase of plasma clearance (Robert *et al.*, 1982). Recent *in vitro* concentration × time studies using NCI-H69 cells (Twentyman & Fox, in preparation) indicate that the concentration × time of each phase of the patient plasma curve lies within the range able to cause significant effects on cell growth. At the present time, therefore, determinations of relative potency based on *in vitro* testing must be regarded as general indicators rather than precise quantitative predictions of likely *in vivo* effects.

Our finding that Ro 31-1215 shows little loss of activity in ADM-resistant cells is of considerable potential importance. It is widely accepted that the development of resistance to ADM is a significant clinical problem. Most anthracyclines that have been studied have been found to lose activity in ADM-resistant cells. A mouse fibrosarcoma line resistant to ADM was also resistant to daunorubicin and to mAMSA (Giavazzi *et al.*, 1983) and similar conclusions were reached by Schabel *et al.* (1983) using an ADM-resistant subline of P388 leukaemia. For aclacinomycin A (ACL), however, little loss of activity appears to occur in ADM-resistant lines (Tsuruo *et al.*, 1983; Hill *et al.*, 1985; Twentyman *et al.*, 1985). In addition, retention of activity in an ADM-resistant line of L5178Y lymphoma was seen for 4 anthracyclines (including 4'deoxyadriamycin) by Hill *et al.* (1985). However, the ADM-resistance factor for this line was low. Our own studies for 4'deoxyadriamycin (Twentyman *et al.*, 1985) show as great a loss of activity as that seen for ADM.

The data presented in this paper for Ro 31-1215 indicate that it is of similar efficiency to ACL in overcoming ADM-resistance (Twentyman *et al.*, 1985). The subline H69/LX of small cell lung cancer line NCI-H69, is resistant to ADM by a factor which varies between 4 and 30 in individual experiments, but is not resistant to either ACL or Ro 31-1215. The subline H69/LX4 (resistance factor for ADM = 40–200) is resistant to ACL and Ro 31-1215 by 2–4 ×. We therefore believe that Ro 31-1215 is as good a candidate as ACL for an anthracycline with retained activity in ADM-resistant cells. Our studies on cellular content of ADM and Ro 31-1215 during prolonged incubation indicate that cellular pharmacokinetic differences may be involved in these relative resistance characteristics. It is interesting that EMT6 cells made resistant to Ro 31-1215 by growth in the drug show a higher resistance factor for ADM than they do for Ro 31-1215 itself. This may indicate that the mechanism of resistance in these cells is the same as that in cells made resistant by growth in ADM. Additional studies using compounds synthesised by Roche Products (Scott *et al.*, 1985; 1986) have determined that several 9-methyl and 9-ethyl substituted 4-demethoxy anthracyclines also show retention of activity in ADM-resistant cells. This may indicate the prime importance of a 9-alkyl substitution in conferring such a property. The fact that ACL also has a 9-alkyl group would support this proposition.

We are currently carying out detailed studies in animal systems designed to compare directly the 3 novel anthracyclines reported in this paper with ADM in terms of therapeutic efficiency. Studies into the relative cardiotoxicity of the compounds are also in progress.

### References

BAILLIE-JOHNSON, H., TWENTYMAN, P.R., FOX, N.E. & 6 others (1985). Establishment and characterisation of cell lines from patients with lung cancer (predominantly small cell carcinoma). *Br. J. Cancer*, **52**, 495.

DAVIS, H.L. & DAVIS, T.E. (1979). Daunorubicin and Adriamycin in cancer treatment: An analysis of their roles and limitations. *Cancer Treat. Rep.*, **63**, 809.

GIAVAZZI, R., SCHOLAR, E. & HART, I.R. (1983). Isolation and preliminary characterization of an adriamycin-resistant murine fibrosarcoma cell line. *Cancer Res.*, **43**, 2216.

HARTMANN, H.R., BROADHURST, M.J., THOMAS, G.J. & MARTIN, J.A. (1985). Antitumour effect of new anthracyclines in mice. *Br. J. Cancer*, **52**, 422 (abstract).

HILL, B.T., DENNIS, L.Y., LI, X.T. & WHELAN, R.D.H. (1985). Identification of anthracycline analogues with enhanced cytotoxicity and lack of cross-resistance to adriamycin using a series of mammalian cell lines in vitro. *Cancer Chemother. Pharmacol.*, **14**, 194.

HUBBARD, S.M., BARKER, P. & YOUNG, R. (1978). Adriamycin therapy for advanced ovarian carcinoma recurrent after chemotherapy. *Cancer Treat. Rep.*, **62**, 1375.

KAYE, S. & MERRY, S. (1985). Tumour cell resistance resistance to anthracyclines – A review. *Cancer Chemother. Pharmacol.*, **14**, 96.

MINOW, R.A., BENJAMIN, R.S. & GOTTLIEB, J.A. (1975). Adriamycin (NSC 123127) cardiomyopathy – An overview with determination of risk factors. *Cancer Chemother. Rep.*, **6**, 195.

NAFF, M.B., PLOWMAN, J. & NARAYANAN, V.L. (1982). Anthracyclines in the National Cancer Institute Program. In *Anthracycline Antibiotics*, El Khadem, H.S. (ed) p. 1. Academic Press: New York.

ROBERT, J., ILLIADIS, A., HOERNI, B., CANO, J-P., DURAND, M. & LAGARDE, C. (1982). Pharmacokinetics of adriamycin in patients with breast cancer: Correlation between pharmacokinetic parameters and clinical short-term response. *Europ. J. Cancer Clin. Oncol.*, **18**, 739.

SCHABEL, F.M., SKIPPER, H.E., TRODER, M.W., LASTER, W.R., GRISWOLD, D.P. & CORBETT, T.H. (1983). Establishment of cross-resistance profiles for new agents. *Cancer Treat. Rep.*, **67**, 905.

SCHWARTZ, H.S. (1973). Fluorimetric assay for daunomycin and adriamycin in animal tissues. *Biomed. Med.*, **7**, 396.

SCOTT, C.A., WESTMACOTT, D., BROADHURST, M.J., THOMAS, G.J. & HALL, M.J. (1985). 9-alkyl anthracyclines. Absence of cross-resistance in a human cell line. *Br. J. Cancer*, **52**, 423 (abstract).

SCOTT, C.A., WESTMACOTT, D., BROADHURST, M.J., THOMAS, G.J. & HALL, M.J. (1986). 9-alkyl anthracyclines. Absence of cross-resistance to adriamycin in human and murine cell cultures. *Br. J. Cancer*, **53**, this issue.

SKOVSGAARD, T., DANO, K. & NISSEN, N.I. (1984). Chemosensitizers counteracting acquired resistance to anthracyclines and vinca alkaloids in vivo. A new treatment principle. *Cancer Treat. Rev.*, **11** (Suppl. A), 63.

TSURUO, T., IIDA, H., TSUKAGOSHI, S. & SAKURAI, Y. (1983). Potentiation of vincristine and adriamycin effects in human haemopoietic tumor cell lines by calcium antagonists and calmodulin inhibitors. *Cancer Res.*, **43**, 2267.

TWENTYMAN, P.R., FOX, N.E., WRIGHT, K.A. & BLEEHEN, N.M. (1986). Derivation and preliminary characterisation of adriamycin resistant lines of human lung cancer cells. *Br. J. Cancer*, **53**, April.

ZENEBERGH, A., BAUVAIN, R. & TROUET, A. (1982). Cellular pharmacokinetics of aclacinomycin A in cultured L1210 cells. Comparison with daunorubicin and doxorubicin. *Cancer Chemother. Pharmacol.*, **8**, 243.